# Numerical Methods and Applications in Biomechanical Modeling 2014

**DOI:** 10.1155/2015/768652

**Published:** 2015-09-01

**Authors:** Eduardo Soudah, Eddie Y. K. Ng, Zhonghua Sun, Spandan Maiti

**Affiliations:** ^1^Centre Internacional de Mètodes Numèrics en Enginyeria (CIMNE), Universitat Politècnica de Catalunya, C/Gran Capitan, s/n, 08034 Barcelona, Spain; ^2^School of Mechanical and Aerospace Engineering, Nanyang Technological University, Singapore 639798; ^3^Department of Medical Radiation Sciences, Curtin University, Perth, WA 6102, Australia; ^4^Department of Bioengineering, University of Pittsburgh, Pittsburgh, PA 15261, USA

Numerical methods and applications in biomechanical modeling have continued to play increasingly important roles in biomedical research and applications. This special issue, following the very successful one in 2013, provides a snapshot of the emerging biomedical applications and research. The main focus of this special issue was on the interface between numerical methods and biomedical applications especially for cardiovascular dynamics and biomechanics problem in the human body. The goal of this special issue was to bring together experts in related fields of computational biomedical engineering like multiscale flow modeling (3D, 1D, and 0D models), blood flow propagation, boundary conditions, fluid-solid coupling, inverse problems in biomechanics, high-performance computing of multiphysics discretization schemes, cardiovascular biomechanics, and porous media. Then, the details of these papers are summarized as follows.

The work of M. Klous et al. attempts to compare ankle and knee joint loading at the steering leg between carved ski and snowboard turns. The authors showed higher forces along the longitudinal axis in skiing and similar forces for skiing and snowboarding in anterior-posterior and mediolateral direction. This study can help the clinician to improve understanding of how forces are distributed in the ankle and knee joint when these sports are done.

X. Liu et al. present the deformation and haptic feedback of soft tissue in virtual surgery based on a liver model by using a force feedback device. This paper introduces a kind of mesh-free method for deformation simulation of soft tissue and force computation based on viscoelastic mechanical model and smoothed particle hydrodynamics (SPH). The results reveal that SPH methodology is suitable for simulating soft tissue deformation and force feedback calculation, and SPH based on dynamic local interaction area has a higher computational efficiency significantly compared with the usual SPH.

A. Belwadi and K. H. Yang show an interesting paper about how the occupant-seating position, bumper profile height, and the principal direction of force of impact play a crucial role in the generation of strain and pressure in the aorta and a potential injury mechanism responsible for traumatic rupture of the aorta in automobile crashes. In their study, 16 design of computer experiments were carried out with varying levels of principal direction of force, impact velocity, impact height, and impact position of the bullet vehicle combined with occupant-seating positions in the case vehicle to determine the effects of these factors on aortic injury. Simulation results showed that, in simulated near side left lateral crashes, peak average maximum principal strain mainly took place in the isthmus of the aorta. Their design of computer experiments using finite element vehicle models has identified the key factors responsible for aortic injury.

N. Mijailovic et al. combine within biomechanical model different sensor measurements to determine the knee cartilage deformation ratio and the knee cartilage stress distribution to predict when it is necessary to perform surgery on a patient. The model includes the impact of ground reaction forces, contact force between femur and tibia, patient body weight, ligaments, and muscle forces. Despite introduction of a new approach and presentation of some preliminary findings, their proposed method shows great potential for preoperative and postoperative surgical planning and treatment of patients with knee injuries.

The paper by M. Jahangiri et al. compares different turbulent models over a stenosed artery considering an elastic wall. The results were compared with those of the laminar flow assumption and the rigid artery wall and show the effects of turbulent blood flow over the velocity profiles.

F. Schellenberg et al. present a review of existing computational techniques to determine muscle forces in the lower limbs during strength exercises* in vivo* and discuss their potential for uptake into sports training and rehabilitation. The review introduces the different computational techniques and outlines their advantages and disadvantages for the informed usage by nonexperts. With sufficient validation and widespread application, muscle force calculations during strength exercises* in vivo* are expected to provide biomechanically based evidence for clinicians and therapists to evaluate and improve 20 training guidelines.

R. Rockenfeller et al. compare two models of mammalian striated muscle activation dynamics proposed by Hatze and Zajac and perform a sensitivity analysis for investigating the influence of model parameters on the solution of the mathematical equations. The authors also used a global sensitivity analysis approach to factor in finite ranges of parameter values. The authors demonstrate that the findings of global sensitivity analysis must be treated with caution because the whole dynamics of a system is condensed to a single average function per whole parameter range.

The work of S. Khalafvand et al. studies the blood flow characteristics in the normal left ventricle. The authors show the vortices produced (generation and growth) and their correlation with flow acceleration and deceleration during the mitral valve opening and closing. This work can help the cardiologist to understand how the vortices are produced in the heart movement, thus contributing to understanding of pathogenesis of cardiac disease.

The objective of the work by Þ. Pétursson et al. describes a novel preliminary methodology for patient evaluation before total hip replacement surgery as a first step towards creating a patient-specific, presurgical application for determining the optimal prosthesis procedure. Ten patients were studied using finite element analysis and bone mineral density to estimate the status of hip before surgery; after that a fracture risk index is defined and compared with the patient's age, sex, and average proximal bone mineral density. Findings of this study showed a high degree of variability between patients grouped according to implant procedure, with age and gender being the poor indicators for determining total hip replacement procedure. Their results could be used as a basis to develop a clinical database for correlating bone mineral density and fracture risk index to total hip replacement patient outcomes.

G. Saborit and A. Casinos present a mathematical model to predict the optimum gradient for a minimum energetic cost. The model focuses on the variation in mechanical energy during gradient walking. The authors show that kinetic energy plays a marginal role in low speed gradient walking. Consequently, the optimal negative gradient depends on the individual step length.

In this special issue, we have provided examples of recent progress in computational and mathematical methods in biomedicine, for the benefit of students, researchers, healthcare professionals, and teachers.

